# Image and fractal analysis as a tool for evaluating salinity growth response between two *Salicornia europaea* populations

**DOI:** 10.1186/s12870-020-02633-8

**Published:** 2020-10-12

**Authors:** S. Cárdenas-Pérez, A. Piernik, A. Ludwiczak, M. Duszyn, A. Szmidt-Jaworska, J. J. Chanona-Pérez

**Affiliations:** 1 Chair of Geobotany and Landscape Planning, Faculty of Biological and Veterinary Sciences, Nicolaus Copernicus University, Lwowska 1, 87-100 Toruń, Poland; 2 Chair of Plant Physiology and Biotechnology, Faculty of Biological and Veterinary Sciences, Nicolaus Copernicus University, Lwowska 1, 87-100 Toruń, Poland; 3 Departamento de Ingeniería Bioquímica, Escuela Nacional de Ciencias Biológicas, Instituto Politécnico Nacional, Av. Wilfrido Massieu, Esq. Manuel L. Stampa s/n, 07738, Gustavo A. Madero, Ciudad de México, Mexico

**Keywords:** Halophyte, Fractal architecture, Colour analysis, Morphometry, Genetic analysis

## Abstract

**Background:**

This study describes a promising method for understanding how halophytes adapt to extreme saline conditions and to identify populations with greater resistance. Image and colour analyses have the ability to obtain many image parameters and to discriminate between different aspects in plants, which makes them a suitable tool in combination with genetic analysis to study the plants salt tolerance. To the best of our knowledge, there are no publications about the monitoring of halophytic plants by non-destructive methods for identifying the differences between plants that belong to different maternal salinity environments. The aim is to evaluate the ability of image analysis as a non-destructive method and principal component analysis (PCA) to identify the multiple responses of two *S. europaea* populations, and to determine which population is most affected by different salinity treatments as a preliminary model of selection.

**Results:**

Image analysis was beneficial for detecting the phenotypic variability of two *S. europaea* populations by morphometric and colour parameters, fractal dimension (FD), projected area (A), shoot height (H), number of branches (B), shoot diameter (S) and colour change (ΔE). S was found to strongly positively correlate with both proline content and ΔE, and negatively with chlorophyll content. These results suggest that proline and ΔE are strongly linked to plant succulence, while chlorophyll decreases with increased succulence. The negative correlation between FD and hydrogen peroxide (HP) suggests that when the plant is under salt stress, HP content increases in plants causing a reduction in plant complexity and foliage growth. The PCA results indicate that the greater the stress, the more marked the differences. At 400 mM a shorter distance between the factorial scores was observed. Genetic variability analysis provided evidence of the differences between these populations.

**Conclusions:**

Our non-destructive method is beneficial for evaluating the halophyte development under salt stress. FD, S and ΔE were relevant indicators of plant architecture. PCA provided evidence that anthropogenic saline plants were more tolerant to saline stress. Furthermore, random amplified polymorphic DNA analysis provided a quick method for determining genetic variation patterns between the two populations and provided evidence of genetic differences between them.

## Background

Salinity is nowadays an important environmental problem disturbing plant growth. It has been reported by the [17] that soil salinity has negative impacts on agricultural production, and in particular pollutes natural resources, affecting the balance of ecosystems. Meanwhile, Nelson and Mareida [41] reported in 2001 that more than 10 million ha of irrigated land is excluded from use in production due to high salinity. In this sense, halophytic plants are effective at salt adaptation, as they have a suitable mechanism to grow under salt stress and could be beneficial in the bioremediation of saline soils. The study of halophytes such as *Salicornia europaea* can help to understand how this type of plant adapts to the extreme conditions of saline areas and to select those that are best adapted. *Salicornia* belongs to the *Chenopodiaceae* family and is one of the most salt tolerant genotypes, capable of growing under hyper-saline drainage water. Some studies have reported that its growth and net photosynthetic rate are stimulated rather than inhibited under 100 to 400 mM NaCl [6, 20, 33]. However, under high extreme salinity conditions, *Salicornia* experiences modifications in its physiology, cell morphology and biochemistry. The biological effects of salt stress are very different and may include morphological changes such as variation in height, projected area, shoot thickening, plant branching and foliage complexity. They may also include plant colour modification due to a reduced photosynthesis that affects nutrient loss, biomass and hydric balance [40]. Among the few available morphological traits in the genus *S. europaea,* most are extremely variable within species which can probably be attributed to high levels of plasticity or biological adaptations under different environmental conditions [38]. A field experiment performed by Piernik [43] with a Vernier calliper evaluated the shoot height as well as manually identifying the number of shoots, and demonstrated morphological differences between populations growing under different soil salinities. Hairmansis et al. [21] developed a phenotype image analysis as a non-destructive technique for monitoring rice traits under salinity stress. It was concluded that image analysis has the capability to obtain several parameters from - images and to discriminate between the different aspects of salt stress, making it a suitable tool for physiological studies. It was also stated that the image analysis combined with genetic analysis is a useful method for explaining the main processes that influence salinity tolerance in plants. In this context, − recent studies have been looking for simple, accurate and non-destructive methods to evaluate how abiotic stressors affect plants’ growth [7, 19, 30, 32]. Regarding plant architecture, fractal dimension has been proven to be a good indicator for analysing plant foliage changes due to salinity. Some studies have analysed plants’ irregularity by calculating their fractal dimension [18]. Therefore, this parameter has relevance in the study of plant foliage architecture since it can describe the way that plants physically adapt under abiotic stressors, as well as serving as a predictor of plant biomass [11, 25]. Plant colour study by image analysis technique has been used in other studies for different purposes. Karcher and Richardson [26] quantified turfgrass colour through image analysis in order to make comparisons between turf sites. Ma et al. [34] applied colour analysis in leaf images by using image pre-processing technique for identifying deficiencies and excess nitrogen content in soybean leaves. However, to the best of our knowledge, no studies have been published using colour analysis as an indicator to evaluate salt stress in plants.

When plants are exposed to high salinity, they induce a reduced stomatal conductance as a strategic mechanism to decrease the net uptake of salt ions and to conserve water in the plant, causing a mesophyll thickening of the shoot [10]. The lower stomatal conductance mechanism leads to the generation of reactive oxygen species (ROS) while at the same time CO_2_ fixation is reduced, inducing a photosynthetic decrease, which is reflected in changes of the plant pigments due to the reduction in chlorophyll content. The capability of *Salicornia* to manage salt stress effects can be associated with the scavenging of ROS such as O_2_, H_2_O_2_ and OH [37, 48]. Until now, the majority of studies have tested plant salt adaptation through destructive and slow screening techniques in order to measure different morphological traits. Consequently, these conventional techniques are not suitable to measure in situ dynamic responses in plant growth during salt stress. However, sampling in real-time may be done in field conditions. Recent progress in phenotype image analysis have put emphasis on the non-destructive evaluation of salinity responses of plants over time and this allows the plant biomass to be determined and morphometry to be measured without affecting the whole plant [21, 24]. Currently, there is no publication on the monitoring of halophytic plants by non-destructive methods, especially for identifying the differences between plants that belong to different maternal salinity environments. Therefore, in this study, we aim to evaluate the ability of non-destructive methods such as image and colour analysis, fractal dimension as a quantitative measure of plant development and complexity under salinity, as well as principal component analysis (PCA) to identify the multiple responses of two *S. europaea* populations from different salinity sites. It is also the aim of this paper to determine which are the most affected by different salinity treatments as a preliminary model of selection from each sample, as it was hypothesized that non-destructive methods are able to efficiently determine if *S. europaea* populations from different sites (natural and anthropogenic) can adapt to salinity differently.

## Methods

### Plant materials, growth conditions and salt treatments

Soil samples with *S. europaea* seeds were collected at two sites representing a natural and an industrial saline area in Poland. The first site is supported with natural brine in the health resort of Ciechocinek (C) (52°53′N, 18°47′E). Natural salinity in this place is related to salt springs associated with Zechstein salt stratums [44]. The second site is located in the vicinity of a soda factory in the town of Inowrocław-Mątwy (I) (52°48′N, 18°15′E), with salinity at this site linked to waste from soda production [45, 47]. The first site is characterised by high soil salinity *ca* 100 dS/m (~ 1000 mM NaCl) [47, 50], with this type of soil salinity described as chloride (Cl^−^:SO_4_^2−^ > 2.5) with dominant cations: Na> > Ca > Mg > K and anions: Cl> > SO_4_ > HCO_3_ [44]. The second site is characterised by a lower salinity of *ca* 55 dS/m (~ 550 mM NaCl) [47, 50]. The type of soil salinity is also chloride, with dominancy of cation: Ca > Na> > Mg > K and anion: Cl> > SO_4_ > HCO_3_ [44]. The distance between C and I sites is ca 50 km, with both seeming to be fairly isolated from each other. *S. europaea* seeds were collected in October 2018 and were sterilised with bleach diluted in water (30%). The seeds were then germinated in the growth chamber in Petri dishes (Ø 7 cm) with a piece of filter paper and 5 ml of distillate water. Once the seeds germinated, they were planted in individual pots (height: 5.3 cm and diameter: 5.5 cm) with a sterile substrate of vermiculite and sand in a ratio of 1:1, with an experimental unit per pot and 12 seedlings for each salt treatment. Before planting, each group of 12 pots was located on individual trays lacking drainage, and were saturated at their full capacity with solutions of 0, 200, 400, 800 and 1000 mM NaCl (ca 500 ml of solution for 12 pots with the substrate) [46]. The plants were grown in a growth chamber with day/night (25/20 °C) photon flux density of 1000 mmol m^˗2^ s^˗1^, relative humidity of 50–60% and a photoperiod of 16/8 h (light/dark). Seedlings were irrigated through pouring distillate water in the tray for up to 21 days. They were then watered for 30 days with an equal amount of Hoagland’s solution every 2 days to ensure homogeneity of salinity and nutrient supply. In total, 120 plants (12 plants × 5 treatments × 2 populations) were cultivated, and, therefore, a complete randomized design with a factorial design 2^5^ was used, which included a total 120 samples (12 plants × 5 treatments × 2 populations) with 12 response variables. After 2 months of development, morphometric and colour parameters were estimated in 12 samples while proline, hydrogen peroxide, chlorophyll and carotenoid contents per triplicate were determined (plants were randomly selected). The voucher specimen of the plant material has been deposited in a publicly available herbarium of the Nicolaus Copernicus University in Toruń (Index Herbarium code TRN), deposition number not available. (Dr. hab. Agnieszka Piernik, prof NCU undertook the formal identification of plant species and permission to work with the seeds was provided by the Regional Director of Environmental Protection in Bydgoszcz, WPN.6205.159.2014.KLD, WPN.6205.69.2015.KLD, WPN.6205.44.2016.KLD).

### Morphometric and colour analysis

The size and shape of the plants were characterised by images obtained with a Sony digital camera (13 MP, f/2.0, 1/3″, 1.12 μm, focal length 3.79 mm, with autofocus). After 2 months, samples (the entire plants from the pots) were placed inside a photography light box PULUZ (PU5060, HITSAN, China) equipped with two 30 W, 5500 K integrated LED lights which can soften and reflect light and eliminating glare, while the box wall material works as a lighting diffuser generating homogeneous light on the sample. The camera was located at a distance of 50 cm from the samples, and the same light and distance conditions were used for capturing the aerial part of the plants. The images were captured in 12 replicates per treatment for the C and I populations. The images were obtained in RGB and stored in. TIFF format at 4160 × 3120 pixels. The images were converted to greyscale and then to binary images by manual segmentation (threshold from 135 to 240) from cropped greyscale images of individual plants. Finally, the shape and size of the plants were obtained from the binary images. All steps of image analysis were performed in ImageJ v. 1.47 software (National Institutes of Health, Bethesda, MD, USA). The projected area (A) was calculated through the number of pixels inside the borderline, while the shoot diameter (S) was determined by the horizontal distance between the two extremes of the middle segment of the shoot. The number of branches (B) was obtained through the total count of branches per individual, and shoot height (H) corresponds to the distance from the base to the apical part. Furthermore, fractal dimension (FD) was used to evaluate the structural shape of growth, and has been used to analyse the complexity of biological samples in many studies [12, 13]. In the present study, FD was evaluated by means of the fractal box count plugin in ImageJ, where higher FD values correspond to complex images. The values range between 1 and 2, with values near 1 indicating a low irregularity, while values near 2 indicate a more irregular or fractal plant structure, meaning that the plants tend to fill bi-dimensional space more effectively.

The colour change analysis during the salt treatment of plants was carried out according to the methods described by Cárdenas-Pérez et al. [14]. Previous studies concluded that the CIELab space is suitable for the analysis of biological sample colour [35]. The complete plant image (without root) was used to evaluate the colour change of each plant. The values of the pixels on the image of the plant shoots were transformed into CIELab coordinates, a* (green to red) and b* (blue to yellow) and L* (luminosity). The conversion plugin was used to convert RGB to CIELab (Illuminant D65). Total colour difference (ΔE) was calculated with equation 1:

1 $$ \varDelta E=\sqrt{{\left({\varDelta L}^{\ast}\right)}^2+{\left({\varDelta a}^{\ast}\right)}^2+{\left({\varDelta b}^{\ast}\right)}^2} $$

where ΔL  =  L*-L_0_*; Δa  =  a*-a_0_*, Δb  =  b*-b_0_*; the initial colour parameters correspond to the colour value obtained in the control plants without salt treatment (0 mM).

For the colour comparison among treatments and populations, the ΔE parameter was considered a useful descriptive parameter to evaluate the complete difference of colour in each plant. An additional figure file shows a diagram of image analysis carried out herein [see Additional file 1].

### Biochemical analysis

Proline content (P) was measured in plants according to Abraham et al. [1]. Fresh stem material (500 mg) was pulverised on ice and homogenised in a mortar with 3% aqueous sulfosalicylic acid solution (5 μl/mg fresh plant material). The homogenate was centrifuged at 18,000  ×  g, 10 min at 4 °C, and the supernatant was collected. The reaction mixture was composed of 100 μl of 3% sulfosalicylic acid, 200 μl of glacial acetic acid, 200 μl of acidic ninhydrin reagent and 100 μl of supernatant. Acidic ninhydrin reagent was prepared as described by Bates et al. [8]. P was determined based on the standard curve for proline in the concentration range of 0 to 40 μg/ml. The standard curve equation was y  =  0.0467x - 0.0734, R^2^ = 0.963. P was expressed in mg of proline per gram of fresh weight.

Hydrogen peroxide (HP) levels were determined according to the methods described by Velikova et al. [51]. Stem tissues (500 mg) were homogenised with 5 ml trichloroacetic acid 0.1% (w:v) in an ice bath. The homogenate was centrifuged (12,000  ×  g, 4 °C, 15 min) and 0.5 ml of the supernatant was added to potassium phosphate buffer (0.5 ml) (10 mM, pH 7.0) and 2 ml of 1 M KI. The absorbance was read at 390 nm, and the HP content was given on a standard curve from 0 to 40 mM. The standard curve equation was y  =  0.0188x  +  0.046, R^2^ = 0.987. HP concentrations were expressed in nM per gram of fresh weight.

Chlorophylls (Ch a and Ch b) and carotenoids were extracted from fresh plant stems (100 mg) using 80% acetone for 6 h in darkness, and then centrifuged at 10,000 rpm, 10 min. Supernatants were quantified spectrophotometrically. Absorbances were determined at 646, 663 and 470 nm and the equations 2, 3, 4 were used for calculations according to Lichtenthaler and Welburn [31] when 80% of acetone is used as dissolvent. Total chlorophyll content was calculated as the sum of chlorophyll a and b contents.

2 $$ Cha=\frac{\left(12.21\times {A}_{663}\right)-\left(2.81\times {A}_{646}\right)\times ml\  Acetone}{mg\  sample} $$

3 $$ Chb=\frac{\left(20.13\times {A}_{646}\right)-\left(2.81\times {A}_{663}\right)\times ml\  Acetone}{mg\  sample} $$

4 $$ Carot=\frac{\left(\left(1000\times {A}_{470}\right)-3.27(Cha)-104(Chb)\right)/227\times ml\  Acetone}{mg\  sample} $$

### DNA extraction and RAPD analysis

A complementary genetic analysis was developed as part of an initial attempt to identify the genetic variation patterns among *S. europaea* populations, with a total of 30 individuals of each population ‘in situ’ in the field sampled. The random amplified polymorphic DNA (RAPD) fingerprint method was applied as it has been reported as the fastest and simplest method for investigating genetic variability patterns. Three random primers were selected for the analysis: K01 (5′-CATTCGAGCC-3′), M02 (5′-ACAACGCCTC-3′) and OPB11 (5′-GTAGACCCGT-3′) (Operon Technologies Inc.) based on what has been reported in previous studies [28, 36].

DNA was extracted using CTAB protocol from 100 mg of ground frozen tissue with 1 ml of extracted buffer (CTAB-buffer 20 mg/ml, TRIS-HCL 0.1 M pH 8, NaCl 1.4 M, EDTA 20 mM pH 8 and 0.5% β-mercaptoethanol). Random amplified polymorphic DNA assays were performed in 25 μL total volume containing 2.5 μl of buffer (with 1.5 mM final concentration of MgCl2), 0.5 μl of dNTP (0.2 mM of each dNTP), 0.5 μl of primer (0.1 μM) and 0.625 μl of Taq DNA polymerase (0.65 U) (Eurx, Molecular Biology Products) and 30 ng of DNA. The RAPD-PCR was carried out for 35 cycles consisting of denaturation at 94 °C for 1 min, annealing at 34 °C for 1 min, and extension at 72 °C for 1 min, using an automated thermal cycler. The RAPD fragments were separated by electrophoresis on 1.5% of agarose and visualised by UV. The bands that commonly appeared in each population are defined as monomorphic bands. Conversely, the bands whose presence or absence varied among the plant individuals are considered as polymorphic bands.

### Statistical and multivariate analysis

In order to determine the projection of the effect of salt treatment in plants, a principal component analysis (PCA) was developed using XLSTAT software version 2019.4.1 [52]. For this analysis, twelve variables were used, (projected area A, branch number B, shoot diameter S, height, proline P, hydrogen peroxide HP, chlorophyll a Cha, chlorophyll b Chb, total chlorophyll TC, carotenoids Carot, fractal dimension FD, and total colour difference ΔE), arranged in a matrix with the average values obtained from replicates of each treatment and population. A two-way ANOVA comparing treatments within populations and populations within treatments was conducted for all the results with the Holm–Sidak method using SigmaPlot software version 11.0 [49]. The relationships between variables were performed using a Pearson analysis, while a significance test (Kaisere Meyere Olkin) was performed in order to determine which variables had a significant correlation with each other (α=0.05). Then, a 3D plot was developed using the three principal component factors according to the Kaiser criterion which stated that the factors below the unit are irrelevant. The factorial scores of the PCA from each sample were used to calculate the distance (D) between the two points (populations) under the same treatment P_1_ = (x_1_, y_1_, z_1_) and P_2_ = (x_2_, y_2_, z_2_) in 3D space of the PCA (equation 5).

5 $$ D\left({P}_1,{P}_2\right)=\sqrt{{\left({x}_2-{x}_1\right)}^2+{\left({y}_2-{y}_1\right)}^2+{\left({z}_2-{z}_1\right)}^2} $$

Where x_2_, y_2_, and z_2_ are the three main factorial scores in the PCA corresponding to the evaluated treatment in I and in C. Distances were used to evaluate and determine in which salt treatment the greatest differences between the populations was recorded.

For RAPD analysis, PAST 4.0 software was used to perform a hierarchical agglomerative cluster analysis with the Jaccard’s coefficient as the similarity measure and unweighted pair group method with arithmetic mean (UPGMA) for dendrogram construction [22].

## Results

### Fractal dimension as a measure of plant biomass under different salinity levels

**Fig. 1:**
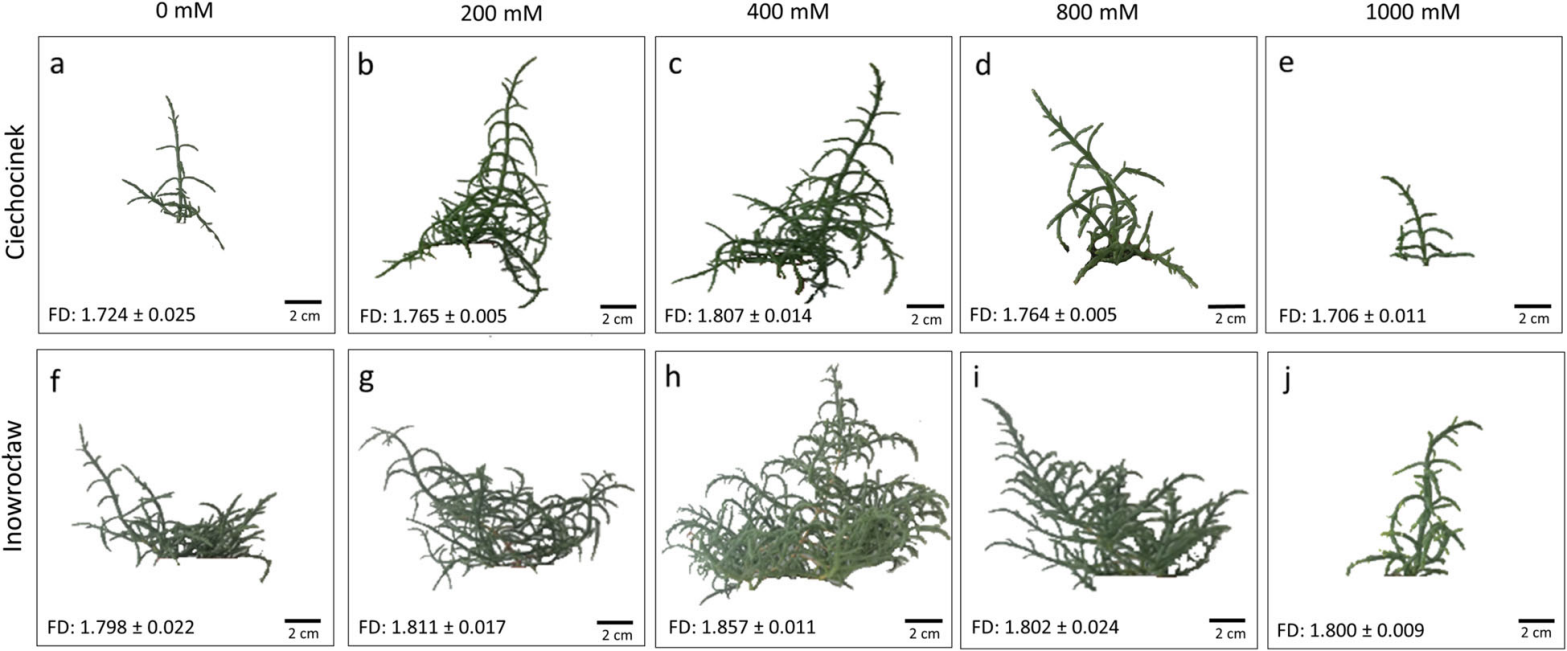
Growth changes and fractal dimensions after two months in *S. europaea*, C (**a**–**e**) and I (**f**–**j**) populations grown under different NaCl concentrations

This study shows the morphometric characteristics of *S. europaea* plants from two different populations that demonstrated a positive effect under moderate salinities 200 and 400 mM NaCl for Ciechocinek and 200, 400 and 800 mM NaCl for Inowrocław, while at the extremes (0 mM and 1000 mM) a decrease in the plant’s biomass was observed. Overall, biomass production was higher in the I population compared to C (Fig. 1). Fractal dimension (FD) was useful for quantitatively characterising the self-similitude properties of plant architecture, with the maximum value reached at 400 mM for C and I. However, in population C, the FD values clearly showed significant differences between salt treatments. Both populations showed significantly different FD values from treatment 0 to 400 mM where an increase of 4.81 and 3.28% was observed for C and I respectively. Moreover, a significant difference was found between the two populations.

### Morphometry analysis in salinity treatments

**Fig. 2:**
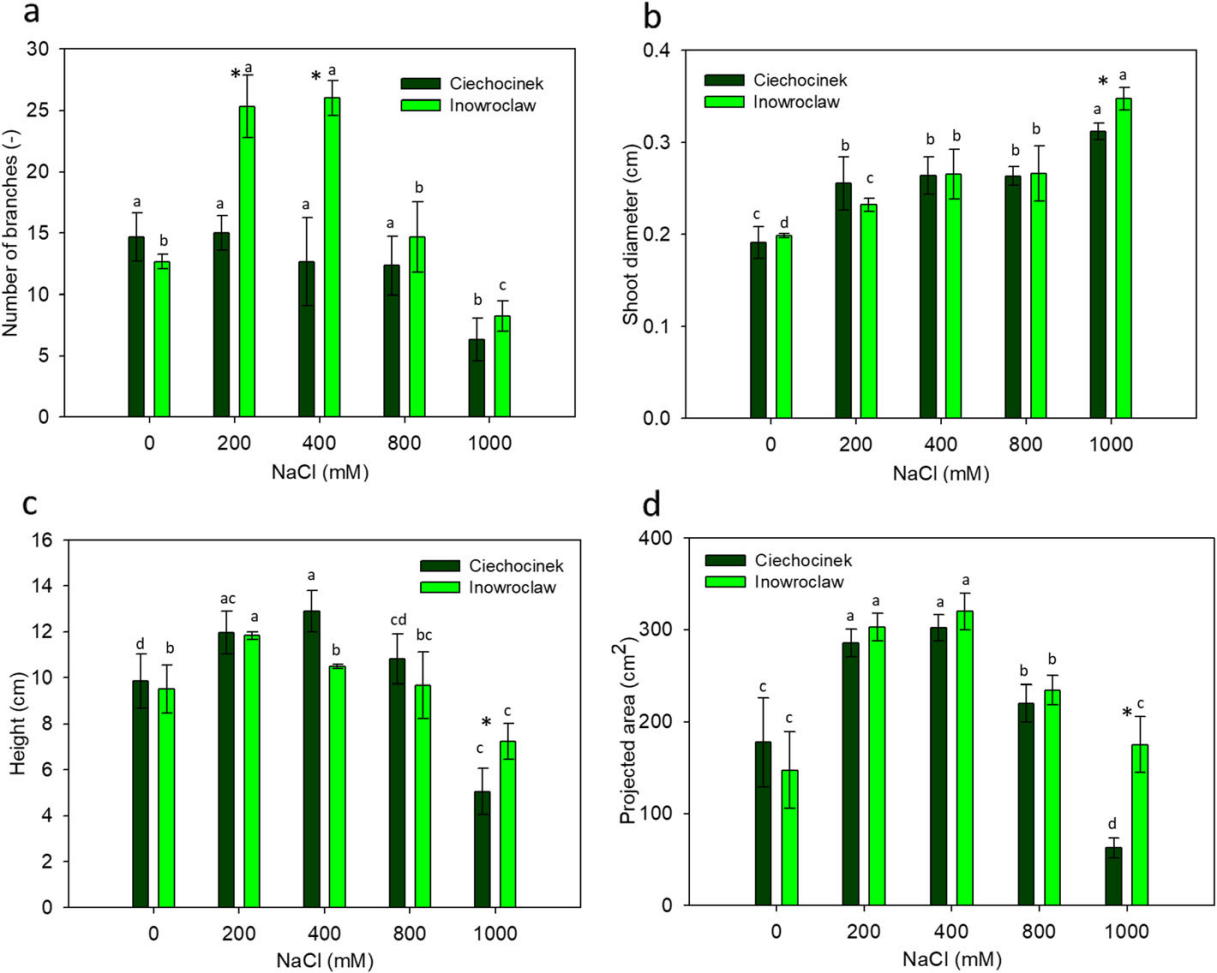
Number of branches (**a**), shoot diameter (**b**) height (**c**) and projected area (**d**) in *S. europaea* populations (Inowrocław and Ciechocinek) under NaCl stress. Means and ± SD of replicates. Different letters indicate significant differences between treatments within each population and * indicates significant difference between populations within treatment (*P* < 0.05)

Each population showed a different behaviour in terms of foliage expansion, which is associated with the significant difference found in the number of branches between both populations within 200 and 400 mM treatment (Fig. 2 a). On the other hand, the projected area and height showed the highest values between 200 and 400 mM of NaCl in both populations, as shown in Figure 2c and d. A significant difference was found between the two populations at 1000 mM NaCl in shoot diameter, height and projected area (Fig 2b,c and d).

### Colour analysis for growth assessment

**Fig. 3:**
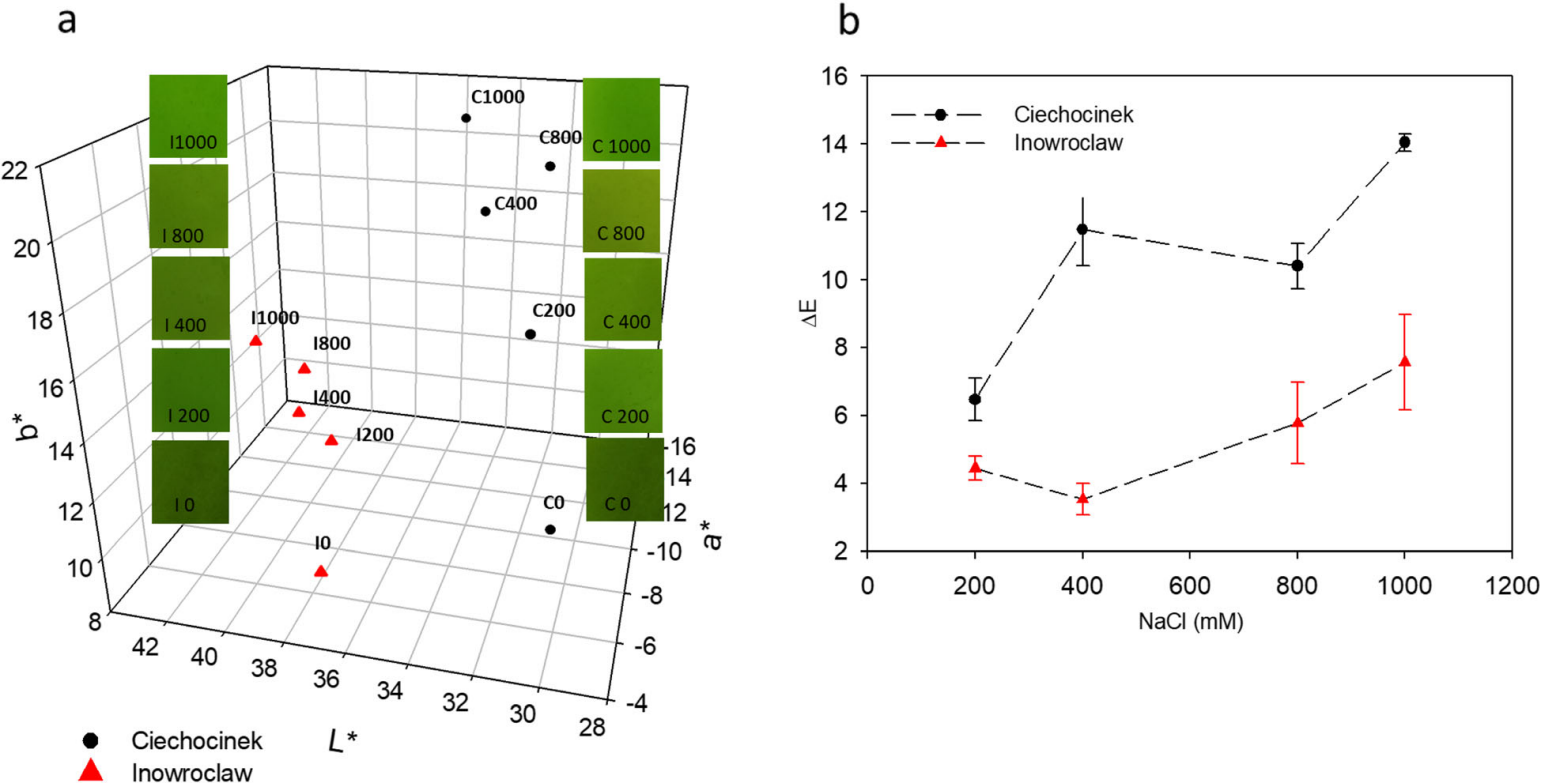
**a**. 3D plot of the colour changes in L* a* and b* parameters of two *S. europaea* populations when subjected to different concentrations of NaCl (representative image crops of each tested plant are shown). **b**. Average values of ΔE (total colour difference of each salt treatment compared to values under 0 mM treatment) in each population. Inowrocław and Ciechocinek bars correspond to ± standard deviation

Colour changes were observed during the assay, and it was interesting that a remarkable difference was observed between plants growing under 0 mM and 1000 mM (Fig. 3). With regard to the L* value, the treatments in the range of 0 to 1000 mM of NaCl increased by 10.91% in I and by 16.67% in C. The a* and b* values show evidence of a decrease and an increase, respectively, between the different salt treatments. This is reflected in the change of a* and b* values from treatment 0 to 1000 mM, with a*decreasing by 66.77% and b* increasing by 60.58% for I, and a* decreasing by 98.19% and b* increasing by 97.36% for C (Fig. 3a). The ΔE value (Fig. 3b) indicates the difference among the samples under 0 mM and under salt treatments. As expected, ΔE increased by 70.11% with salt gradient for I and by 117% for C in the range of 200 mM to 1000 mM. In this sense, the C population showed a higher ΔE increase percentage compared to the I population.

### Relationships between morphometry, colour and biochemical analysis

**Fig. 4:**
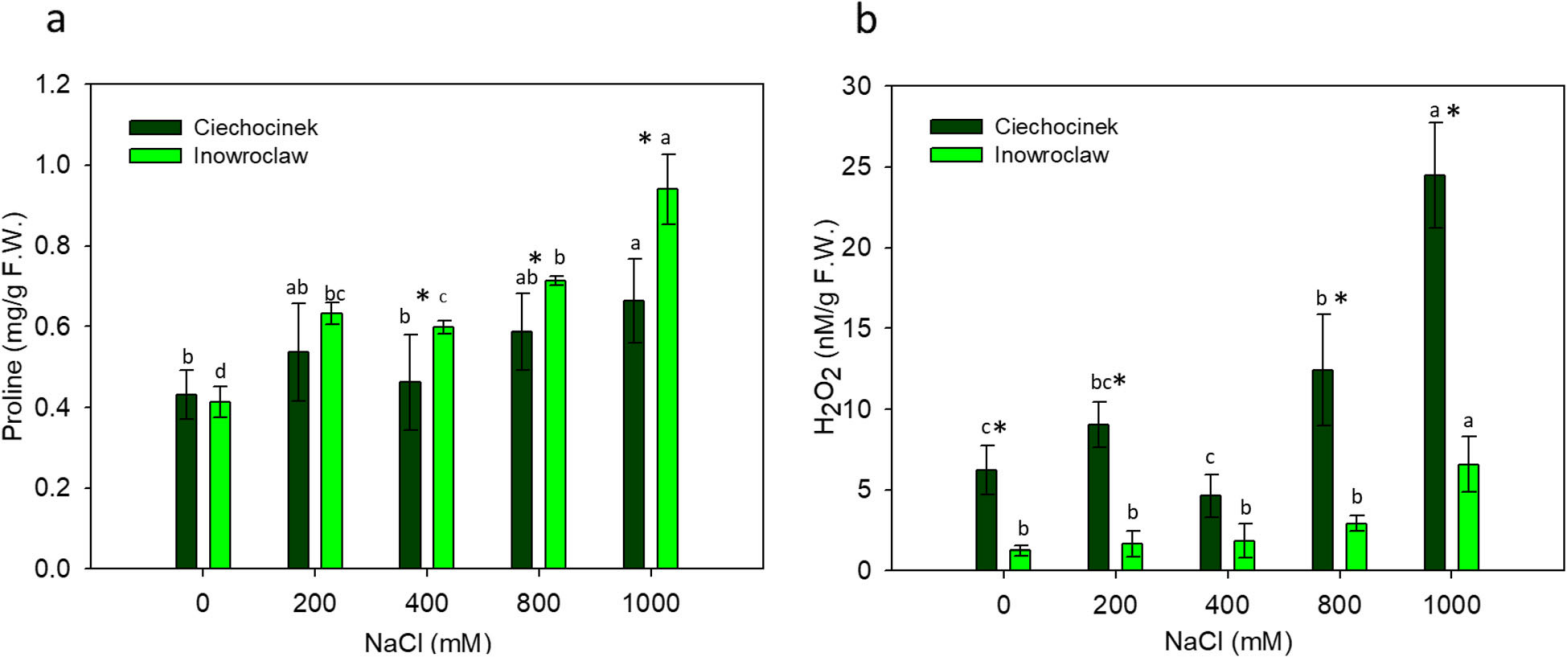
Contents of proline (**a**) and H_2_O_2_ (**b**) in two populations of *S. europaea* (Inowrocław and Ciechocinek) under NaCl stress. Means and ± SD of replicates. Different letters indicate significant differences between treatments within population and * indicates significant difference between populations within treatment (*P* < 0.05)

**Fig. 5:**
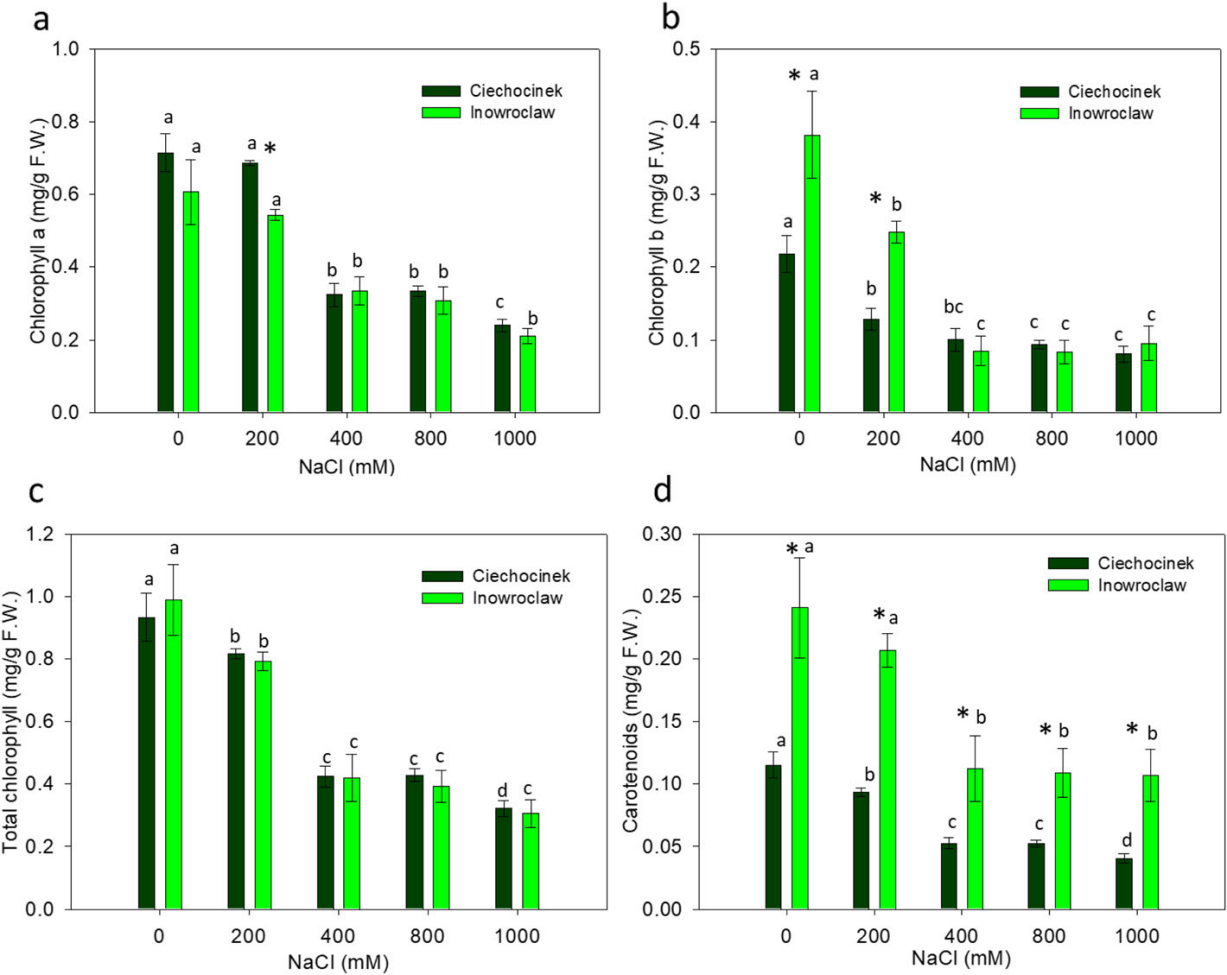
Chlorophyll a (**a**), chlorophyll b (**b**), total chlorophyll (**c**) and carotenoids (**d**) contents in *S. europaea* populations (Inowrocław and Ciechocinek) under NaCl stress. Means and ± SD of replicates. Different letters indicate significant differences between treatments within population and * indicates significant difference between populations within treatment (*P* < 0.05)

P showed an increase with salinity gradient (Fig. 4a). The results show that P was significantly higher in the I population compared to the C population under salt stress, mainly at 400, 800 and 1000 mM. Meanwhile, HP increase is significant only at 800 and 1000 mM NaCl for population C and only at 1000 mM NaCl for population I (Fig. 4b). Chlorophyll a (Ch a), b (Ch b) and carotenoid (Carot), content shows a noteworthy decrease in both populations under NaCl stress (Fig. 5). The chlorophyll content of both populations was significantly different in Ch a at 200 mM and in Ch b at 0 and 200 mM, while there was no significant difference under high salinity (Fig. 5a and b). No significant differences between the two populations were found in total chlorophyll content, but in the case of carotenoid content, significant differences were observed (Fig. 5c and d).

### Principal component analysis (PCA) to evaluate the separation between *S. europaea* populations

**Fig.6:**
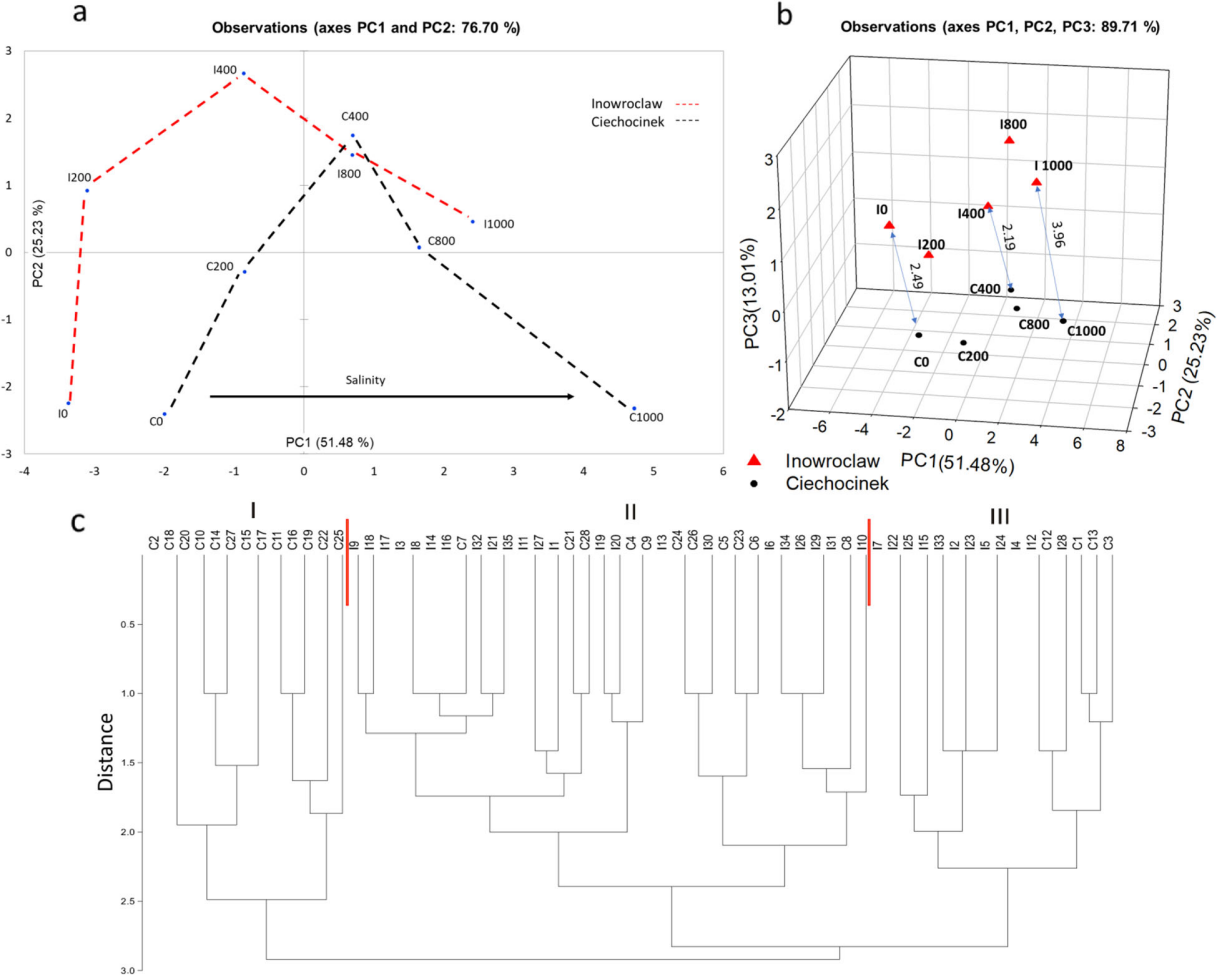
**a**. Scatter plot of the first two principal components with all variables, showing distribution of samples along the gradient of salinity going from left to right. **b**. Three main principal components represented in a 3D plot through showing distances per treatment among both populations. I: Inowrocław, C: Ciechocinek, 0, 200, 400, 800 and 1000 indicate the concentrations in mM of NaCl, and PC the corresponding principal component. **c**. Dendrogram representing the relationships between Inowrocław (I) and Ciechocinek (C) populations of *S. europaea* by random amplified polymorphic DNA analysis (individuals numbered 1–30). Three groups were identified (I, II and III). Jaccard coefficient and UPGMA methods were used

All the variables were evaluated in each population using PCA (Fig. 6a). Figure 6a shows the PC1 and PC2 plots which accurately describe the variability of the samples (76.70%). This plot shows which plants are the most tolerant with regard to saline stress and how they move through the two-dimensional space of the main components, from the negative quadrant of PC1 to the positive quadrant of PC1 as long as salinity increases. The results were also grouped on a 3D plot (Fig. 6b) according to their similarities through the three main factor scores (PC1, PC2 and PC3) which describe the variability of the samples (89.71%) where C plants are more susceptible to salt stress. Factorial scores from the PCA of each sample were used to calculate the distance between the two points under the same treatment P1 = (× 1,y1,z1) and P2 = (× 2,y2,z2) in the 3D space of the PCA (Fig. 6b) for extreme and moderate treatments only (0, 400 and 1000 mM). The comparisons C0 vs I0 (2.49), C400 vs I400 (2.19), and C1000 vs I1000 (3.96) were created in the 3D cartesian axis (x = PC1, y = PC2, z = PC3), with results indicating that the greater the stress, the greater the separation. In addition, a shorter distance is observed at the optimum point (400 mM).

### Random amplified polymorphic DNA (RAPD)

The RAPD analysis of 50 *S. europaea* plants from two populations with three different primers (K01, M02, OPB11) yielded 15 polymorphic bands. This analysis indicated that the M02 and OPB11 primers have the highest number of polymorphic bands (six), while the K01 primer has the lowest number of polymorphic bands (three). Finally, RAPD analysis shows the relationships between the studied populations which are represented by an unweighted pair group method with an arithmetic mean (UPGMA) dendrogram (Figure 6c). Non-typical bands are present for samples in groups II and III, while group I corresponds to bands solely for C (13 samples out of 28).

## Discussion

The higher FD values correspond to a complex and irregular growth pattern of the plants and therefore to an extensive major branching index as well as an optimisation of the space for optimal growth [5, 15], which results in a mechanism of adaptation to support the stress shown in Figure 1. The FD results obtained are in accordance with those obtained by Karamchedu [25] who studied the foliage of various plants and found that the optimal fractal dimension for photosynthetic efficiency is close to 1.85 in plants, while Bayirli et al. [9] studied the FD in *Cercis canadensis L., Robinia pseudoacacia L., Amelanchier arborea (F.Michx.) Fernald, Prunus persica (L.)* as well as others, and concluded that the FD with surface density function could be used as a new approach for the taxonomical study of plants. Such measurements give an overall quantitative degree of the growth and fractal architecture of the plants. On the other hand, fractal analysis has shown to be an efficient tool for describing and predicting ecological patterns at multiple scales. Therefore, our results confirm that fractal analysis used as a measure of plant progress was a useful non-destructive tool for a numerical and simple estimate of the biomass and complexity patterns of branched plants [5] which is able to identify different development patterns between two populations.

Therefore, FD can be an effective measure of the negative and positive development effects between two populations of *S. europaea* under different levels of salinity. The I population showed the highest FD values, especially at the highest salinity treatments with a percentage difference of 5.5%, while both populations have the maximum values (~ 1.850) at 400 mM. According to the results obtained with image analysis for morphological evaluation, *S. europaea* populations appear to have similar behaviour to cope with salinity. However, differences between them are quite visible in each salt treatment such as the height, number of branches, shoot diameter and projected area, which appear higher in the I population, especially at the highest salinity treatment (1000 mM). Furthermore, the I population has the highest values for all the morphological parameters tested, where projected area showed the highest difference at approximately 173%. Therefore, image analysis as a non-destructive method is able to identify differences between the two populations under study.

The novelty of this work is the proof that with image analysis it is possible to obtain more precise, accurate and faster results than with visual methods. For instance, it was possible to observe that the shoot diameter in both populations increases with salinity (a detail that would probably be difficult to obtain using a simple view), which means that this value can also be used as an estimative parameter of the amount of salinity present in the environment where the plant is growing. The I shoot diameter was 11.2% higher than C (Fig. 2 b). The morphometric results from this study are in line with those reported by Piernik [43], who, under a field experiment, demonstrated the inferior growth of *S. europaea* at lower salinity (~ 20 mM NaCl) than for the home zone (~ 200 mM NaCl). The experimental growth optimum for *S. europaea* was described as 300 mM NaCl [39] and under field conditions as 38 dS/m (~ 380 mM NaCl) [44], which is also reflected by this study’s results. Moreover, Szymanska et al. [50] reported differences in situ between the investigated populations. Morphometric parameters were measured by manual inspection with a Vernier calliper and the differences were associated with the environmental conditions and specific microbiomes. Our results prove that under controlled conditions the differences remain the same, even when different salinity levels are taken into account. It is our hypothesis that seeds coming from higher maternal salinity have a genetic makeup in which excessive growth is disadvantageous, although further genetical analysis must be carried out to confirm this hypothesis. El-Keblawy et al. [16] evaluated how the maternal salinity environment affects salt tolerance in *Anabasis setifera* a desert halophyte. They found significantly less germination and salinity tolerance in the population collected from high-saline habitat than in the non-saline population, they attribute this to a lower vigour of the seeds from saline soil. In comparison with previous studies [43, 50], the non-destructive methods provided evidence of the differences in a more efficient and accurate manner.

Colour analysis as a complementary non-destructive method was useful for corroborating that salinity affects the photosynthetic pigment content in *S. europaea.* The changes in the L* parameter can be associated with the change from dark green to bright green in the plants due to the lack of chlorophyll. According to certain studies related to colour change [23], b* goes from +b* yellow direction; ˗b* blue direction so higher b* values are associated with high levels of xanthophylls and a loss of chlorophylls in the chloroplasts. In contrast, negative a* values indicate that the sample is in the green region and positive a* values indicate that the sample is in the red zone. All these changes are a result of the decrease in the dark greenness of the plants and an increase in light green coloration due to the salinity affecting photosynthetic pigments. The I population has a lower ΔE compared to C, with an 85.46% difference between the two populations in the highest salinity treatment. These results are linked to the chlorophyll and carotenoids analyses which show a decrease with the salinity gradient.

The results indicate that the biosynthesis of pigments in the C population was more affected by salinity. According to Witzel (2018), ΔE values above 5 indicate that the colour difference is perceptible to the human eye, which is an important feature for evaluating phenotypic changes quantitatively through colour image analysis as a non-destructive method. Therefore, our hypothesis that non-destructive methods (FD, image and colour analysis) are able to identify differences between populations subjected to different treatments was proved.

Regarding the proline results, it is already known that proline is an osmotic regulator, enzyme denaturation protector and macromolecule assembly stabiliser that allows additional water to be reserved from the environment. This was observed by an increase in succulence allowing water potentials to decrease [4, 29], and this can be physically observed as shoot thickening through image analysis. Our results are in accordance with studies carried out by Akcin and Yalcin [4], Aghaleh et al. [3] and Aghaleh et al. [2] for *S. europaea*. The drastic difference in HP content between two populations can be used to corroborate which is more salt-tolerant. According to Kong-ngern et al. [27], salt-tolerant cultivars showed less hydrogen peroxide content compared to salt-sensitive cultivars, with this study indicating that C is more salt-sensitive when compared to I.

The chlorophyll content of both populations was significantly different at low salinity, while under high salinity there was no significant difference which corroborates our findings obtained through colour analysis. In this sense, it is important to note that Ch b type is an adaptive feature of adapted chloroplasts, while high Ch b content produces an increase in the range of wavelengths absorbed by the chloroplasts, which is attributed as a mode of adaptation when plants are subjected to some abiotic stressor [42]. In this study, the I population showed a statistically significant higher Ch b content compared to population C under 0- and 200-mM treatments.

In PCA it is possible to observe that both populations have a similar tendency when they are subjected to different salt treatments, with both demonstrating good adaptation at 400 mM (Table 1). However, the I population seems to cope better with salinity because under 1000 mM it behaves similarly to C under 800 mM, while at 800 mM, I behaves similarly to C at 400 mM. This suggests that population I is less affected under high salinity. However, according to Szymańska et al. [50] higher activity of *S. europaea* endophytic microorganisms from the more saline site (C) increases the biomass of roots and a higher density of microbial populations influences differences in morphology of the upper part of the plants, such as shorter length of shoots and the number of first order lateral shoots.

The results of the correlation between investigated parameters are of great interest and some have not been reported before, especially the positive correlation between proline and shoot diameter (0.840) (Table 1). Moreover, the inverse correlation between FD and HP is also an interesting finding, with this result suggesting that when the plant is under salt stress, the HP increases and this is reflected in a reduction in the plant complexity or foliage architecture. In addition, there is a high inverse correlation between the S of the plants (the higher the shoot diameter, the higher the plant succulence) and chlorophyll content (Cha, Chb and TC), which means that succulence is lower when chlorophyll pigments are higher. Plants under high salinity tend to store more water due to a lower stomatal conductance, which leads to an increase in shoot diameter while at the same time decreasing photosynthetic pigments by photoinhibition. Furthermore, S has a high positive correlation with ΔE. The biochemical results are in concordance with the morphometric analysis, particularly FD and the colour analysis, which shows that the use of these descriptors is an efficient way of evaluating the growth of plants subjected to saline stress. These descriptors could also be used in the field or in the laboratory as a non-destructive, economic and reliable test which confirms the hypothesis of the work.

**Table 1:** Pearson correlation matrix of the morphometric, colour and biochemical parameters.

Variables	Projected area (A)	Branch num (B)	Shoot diam (S)	Height (H)	Proline (P)	Hydrogen Peroxide (HP)	Chlorophyll a (Cha)	Chlorophyll b (Chb)	Total Chlorophyll (TC)	Carotenoids (C)	Fractal dimension (FD)	Colour difference (ΔE)
A	**1**											
B	**0.733**	**1**										
S	−0.170	−0.415	**1**									
H	**0.880**	**0.719**	−0.530	**1**								
P	−0.159	−0.194	**0.840**	−0.486	**1**							
HP	−0.608	−0.629	0.461	−0.618	0.162	**1**						
Cha	0.141	0.289	**−0.824**	0.455	**−0.673**	− 0.313	**1**					
Chb	−0.168	0.178	**−0.736**	0.221	−0.520	−0.424	**0.682**	**1**				
TC	0.036	0.271	**−0.858**	0.404	**−0.670**	− 0.380	**0.962**	**0.857**	**1**			
C	0.034	0.444	−0.562	0.269	−0.198	**− 0.652**	0.513	**0.876**	**0.691**	**1**		
FD	**0.655**	0.611	0.078	0.443	0.210	**−0.769**	−0.234	0.005	−0.163	0.370	**1**	
ΔE	−0.116	−0.470	**0.716**	−0.264	0.348	**0.714**	**−0.687**	**−0.702**	**− 0.748**	**−0.777**	− 0.265	**1**

Values in bold indicate significance value (except diagonal) at a level of significance α=0.05

Results in Figure 6a allow for the visualisation of population salt tolerance and how both populations move through the bidimensional space, with the advantage being that all the estimated parameters in the PCA are considered. This confirms part of our hypothesis that different populations of the same *S. europaea* species may adapt to salinity differently. Additionally, factorial scores were useful for demonstrating that the highest separation between I and C parameters was found at the highest salinity, indicating that C has more modifications at this salinity.

RAPD analysis showed that the population from I has the highest number of polymorphic bands for the three primers K01, OPB11 and M02 with 3, 4 and 5, respectively. Meanwhile, population C has 2, 4 and 4 polymorphic bands for the same primers. This provides evidence of the genetic difference between populations that might be responsible for the different responses to salinity. However, further deep molecular analysis, such as amplified fragment length polymorphism fingerprinting or next-generation sequencing based methods must be performed for a proper genetic variability and analysis of the different gene expressions, because RAPD is now considered a first-approach fingerprint technique.

## Conclusions

This work shows that image analysis was efficient in evaluating salinity-growth response of *S. europaea* as a non-destructive, simple and economical method. Furthermore, FD proved to be a good indicator of the overall foliage development and was able to identify the differences of biomass production between populations and among salt treatments. This non-destructive method is efficient for quantitatively characterising the complexity of plant architecture. The colour analysis was also an efficient method for determining differences between two populations. Moreover, analyzing the shoot diameter through image analysis showed itself to be a good indicator of succulence as well as salinity, both of which would have been very difficult to detect with the naked eye.

The biochemical analysis proved that non-destructive methods provide a sufficient quantity of accurate results without damaging the plant, as confirmed by the Pearson correlation which highlighted the relationships between non-destructive and conventional parameters. PCA provided evidence that the plants from the anthropogenic saline habitat are more tolerant to saline stress. RAPD provided a quick method for determining genetic variation patterns between the two populations which correlates well with the image analysis. Based on our analysis as a whole, it is clear that our applied methods are able to demonstrate that the two *S. europaea* populations do indeed have different mechanisms of salt adaptation, as well as a positive growth effect under moderate salinities. These results can be used in the future for the selection of resistant plants.

The present results obtained with a non-destructive method is novel in the study of salt resistance plants, meaning that researchers can apply these straightforward, low-cost, accurate and fast methods for future experiments related to plant salinity-development responses.

## Supplementary information

**Additional file 1. **Diagram of image processing for morphometric and colour analysis of *S. europaea.*

## Data Availability

The datasets used and/or analysed during the current study are available from the corresponding author on reasonable request.

## Abbreviations

A: Projected area
B: Number of branches
C: Ciechocinek
Carot: Carotenoids
Ch a: Chlorophyll a
Ch b: Chlorophyll b
DNA-RAPD: Random Amplified Polymorphic DNA
FD: Fractal dimension
H: Shoot height
HP: Hydrogen peroxide
I: Inowrocław
P: Proline
PCA: Principal component analysis
ROS: Reactive oxygen species
TC: Total Chlorophyll
S: Shoot diameter
ΔE: Colour change
