# Supplementary figures and images for: Image and fractal analysis as a tool for evaluating salinity growth response between two Salicornia europaea populations
Source: BMC Plant Biol. 2020 Oct 12;20:467. doi: 10.1186/s12870-020-02633-8 (PMC7549212; doi:10.1186/s12870-020-02633-8)

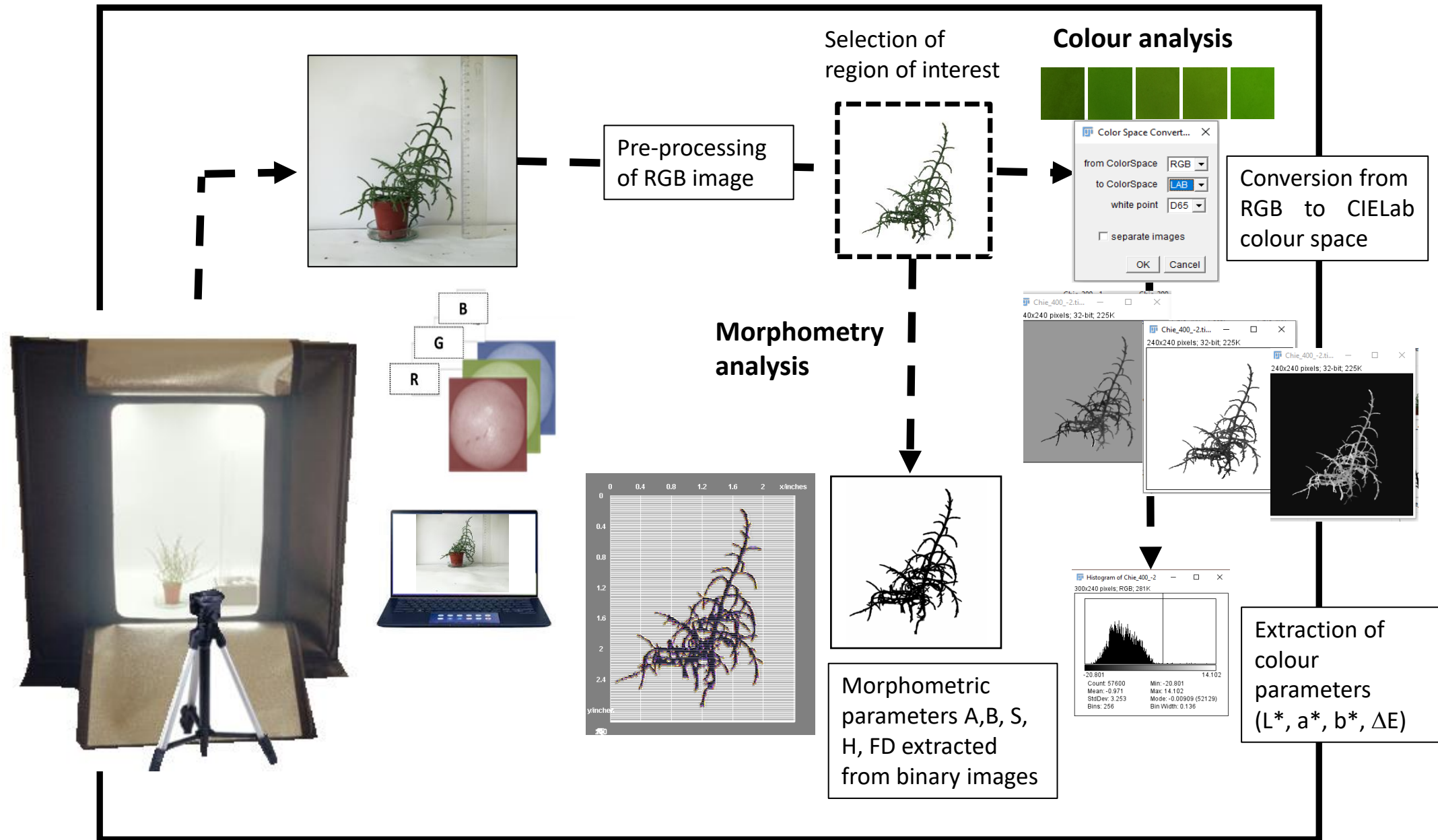

Supplement: Supplementary file 1 — Additional file 1. Diagram of image processing for morphometric and colour analysis of S. europaea. [file 12870_2020_2633_MOESM1_ESM.pdf]
